# Supplementary material for: Knowledge and awareness of cervical cancer in Southwestern Ethiopia is lacking: A descriptive analysis
Source: PLoS One. 2019 Nov 12;14(11):e0215117. doi: 10.1371/journal.pone.0215117 (PMC6850540; doi:10.1371/journal.pone.0215117)
Supplement: S1 File — Each patient was orally interviewed by residents using this standardized questionnaire who then input the information accordingly. The histopathology data (Section IV) was completed by a pathologist. (DOCX) [file pone.0215117.s001.docx]

Annex II Questionnaire

Date:____________

Questionnaire on women with cervical lesions attending the Gynecology Clinic of the Jimma University Specialized Hospital

Consent form (to be read)

Instructions: Fill out and/or circle which apply.

I Socio demographic characteristics

1. Code **____________**

2. Card No __________

3. Age ___________

4. Address_______________

5. Place of residence: a. urban b. semi urban c. rural

6. Occupation: a. Farmer b. Government c. Housewife d. Merchant e. Unemployed f. Other _____________________________

7. Income per year _________ Birr **–**or**-**

income per month ___________Birr

8. Ethnicity: a. Oromo b. Gurage c. Amhara d. Dawro e. Kefa f. Tigre g. Yem h. Others_________

9. Religion: a. Muslim b. Orthodox c. Protestant d.Others____________

10. Literacy status: a. cannot read and write b. read and write informally

c. The highest grade you completed ___________________________________________

11. Marital status: a. Single b. Married c. Divorced d. Widowed

**II History**

1. Parity __________

13. Lifetime number of sexual partners _____

14. Age at first intercourse ________

15. Age at first pregnancy ________

1. Cigarette smoking: a. Yes b. No
2. Did you use any type of contraception? : a. Yes b. No
3. If yes to question **# 17**, which type did you use and for how long?

**Method** **Duration of use**

a. Combined oral contraceptive pill ____________

b. Injectable ____________

c. Implants ____________

d. IUD ____________

e. Sterilization (Tubal Ligation) ____________

f. Condom ____________

g. Natural family planning method ____________

h. Others (specify) _____________ ____________

19. Did you have a history of sexually transmitted diseases? a. Yes b. No

20. If yes to question **# 19**, what diseases? _________________________________

21. Did you have a history of cervical lesions? a. Yes b. No

22. Do you know your HIV status? a. Yes b. No

23. If yes to question **#22**, then what is your status? a. Positive b.Negative

24. Have heard of cervical cancer? a. Yes b. No

25. Do you know the cause of Cervical Cancer? a. Yes b. No

26. If yes to question **#25**, what is the cause? ______________________________

27. If yes to question #**25** what is the mode of transmission?__________________

28. Do you know a clinical method to reduce the morbidity and mortality of cervical cancer? a. Yes b. No

29. If you weren’t ill, would you have been willing to undergo screening?

a. Yes b. No

30. Does your partner have other partners? a. Yes b. No

31. If yes to question **# 30**, then how many? _______________________________

32. Is your partner(s) circumcised? a. Yes b. No c. I do not know

33. Circle the symptom(s) that she has and indicate the duration

**Symptoms Duration**

a. Irregular vaginal bleeding _________________

b. Post coital bleeding _________________

c. Vaginal discharge _________________

d. Pelvic pain _________________

e. Urinary complaints (dysuria, _________________

frequency, urgency, etc.)

**III Physical examination**

34. Type of cervical lesion: a. Fungating b. Ulcerating c. Diffuse/Infiltrative d. Polyp e. Other__________________

35. Does she have contact bleeding on digital examination or speculum examination?

a. Yes b. No

36. Stage: a. Stage I b. Stage IIA c. Stage IIB d. Stage IIIA e. Stage IIIB f. Stage IVA g. Stage IVB

37. HCT/ Hgb ______

38. Other Laboratory finding if any _______________________________________

**IV Histopathology**

39. Pathology lab number__________

40. a. normal epithelium b. inflammation c. regenerative change

41. dysphasia a. mild b.moderate c.severe d. Carcinoma in situ e. Other____________

42. Carcinoma

a. Keratinizing squamous cell carcinoma

b. Large cell non-keratinizing squamous cell carcinoma

c. Small cell carcinoma

d. Adenocarcinoma

e. Others______________

Filled by Dr. _________________

Signature. ____________

Abbreviations:

HPV: Human papilloma virus

HIV: Human immunodeficiency virus

CIN: Cervical intraepithelial lesion

CC: Cervical carcinoma

CD4: Marker for helper T lymphocytes subpopulation

IUD: Intrauterine device
